# Supplementary material for: Association between Workplace Bullying, Job Stress, and Professional Quality of Life in Nurses: A Systematic Review and Meta-Analysis
Source: Healthcare (Basel). 2024 Mar 9;12(6):623. doi: 10.3390/healthcare12060623 (PMC10970563; doi:10.3390/healthcare12060623)
Supplement: Supplementary file 1 [file healthcare-12-00623-s001.zip › Supplementary Table S2.pdf]

Supplementary Table S2. Quality of studies included in the systematic review.

	(Yao et al., 2021)	(Kwak et al., 2020)	(Itzhaki et al., 2018)	(Magnavita, 2014)	(Jiao et al., 2023)	(Peng et al., 2022)	(Kim, Lee & Lee, 2019)	(Choi & Lee, 2017)	(Oh, Uhm & Yoon, 2016)
1. Were the criteria for inclusion in the sample clearly defined?	√	√		√	√	√	√	√	√
2. Were the study subjects and the setting described in detail?	√	√	√	√	√	√			√
3. Was the exposure measured in a valid and reliable way?	√	√	√	√	√	√	√	√	√
4. Were objective, standard criteria used for measurement of the condition?	√	√	√	√	√	√	√	√	√
5. Were confounding factors identified?	√	√	√	√	NA	NA	√	√	NA
6. Were strategies to deal with confounding factors stated?	√	√	√	√	NA	NA	√	√	NA
7. Were the outcomes measured in a valid and reliable way?	√	√	√	√	√	√	√	√	√
8. Was appropriate statistical analysis used?	√	√	√	√	√	√	√	√	√
**Total quality**	Good	Good	Good	Good	Good	Good	Good	Good	Good

NA: not applicable

References

Choi, S.-H. & Lee, H. (2017) Workplace violence against nurses in Korea and its impact on professional quality of life and turnover intention. *Journal of Nursing Management*. 25 (7), 508–518. doi:10.1111/jonm.12488.

Itzhaki, M., Bluvstein, I., Peles Bortz, A., Kostistky, H., Bar Noy, D., Filshtinsky, V. & Theilla, M. (2018) Mental Health Nurse's Exposure to Workplace Violence Leads to Job Stress, Which Leads to Reduced Professional Quality of Life. *Frontiers in Psychiatry*. 9, 59. doi:10.3389/fpsyt.2018.00059.

Jiao, R., Li, J., Cheng, N., Liu, X. & Tan, Y. (2023) The mediating role of coping styles between nurses' workplace bullying and professional quality of life. *BMC Nursing*. 22 (1), 459. doi:10.1186/s12912-023-01624-y.

Kim, Y., Lee, E. & Lee, H. (2019) Association between workplace bullying and burnout, professional quality of life, and turnover intention among clinical nurses. *PloS One*. 14 (12), e0226506. doi:10.1371/journal.pone.0226506.

Kwak, Y., Han, Y., Song, J. & Kim, J. (2020) Impact of emotional labour and workplace violence on professional quality of life among clinical nurses. *International Journal of Nursing Practice*. 26 (1), e12792. doi:10.1111/ijn.12792.

Magnavita, N. (2014) Workplace Violence and Occupational Stress in Healthcare Workers: A Chicken-and-Egg Situation—Results of a 6-Year Follow-up Study. *Journal of Nursing Scholarship*. 46 (5), 366–376. doi:10.1111/jnu.12088.

Oh, H., Uhm, D. & Yoon, Y.J. (2016) Workplace Bullying, Job Stress, Intent to Leave, and Nurses' Perceptions of Patient Safety in South Korean Hospitals. *Nursing Research*. 65 (5), 380–388. doi:10.1097/NNR.0000000000000175.

Peng, J., Luo, H., Ma, Q., Zhong, Y., Yang, X., Huang, Y., Sun, X., Wang, X., He, J. & Song, Y. (2022) Association between workplace bullying and nurses' professional quality of life: The mediating role of resilience. *Journal of Nursing Management*. 30 (6), 1549–1558. doi:10.1111/jonm.13471.

Yao, X., Shao, J., Wang, L., Zhang, J., Zhang, C. & Lin, Y. (2021) Does workplace violence, empathy, and communication influence occupational stress among mental health nurses? *International Journal of Mental Health Nursing*. 30 (1), 177–188. doi:10.1111/inm.12770.
