# Supplementary figures and images for: Association between Workplace Bullying, Job Stress, and Professional Quality of Life in Nurses: A Systematic Review and Meta-Analysis
Source: Healthcare (Basel). 2024 Mar 9;12(6):623. doi: 10.3390/healthcare12060623 (PMC10970563; doi:10.3390/healthcare12060623)

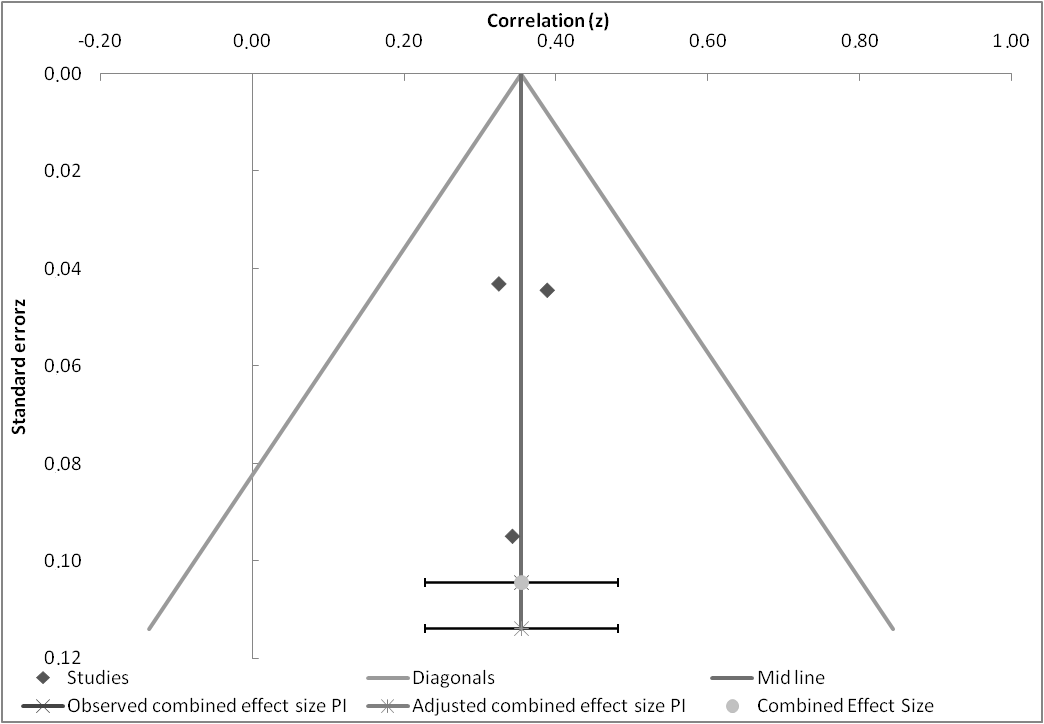

Supplement: Supplementary file 1 [file healthcare-12-00623-s001.zip › Supplementary Figure S1.png]

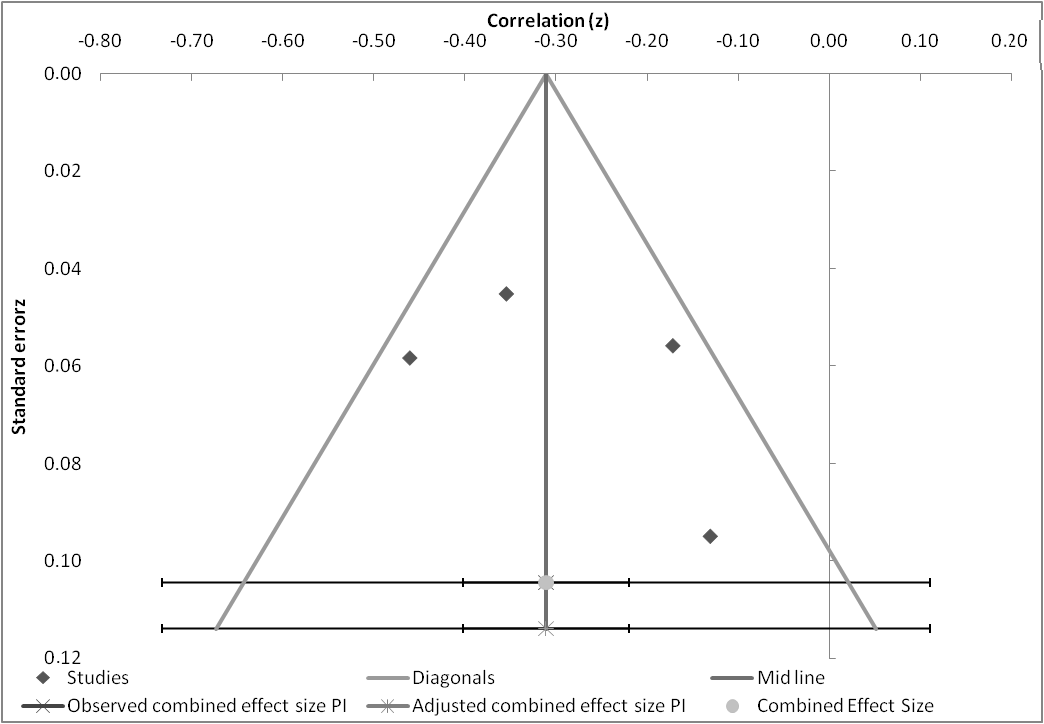

Supplement: Supplementary file 1 [file healthcare-12-00623-s001.zip › Supplementary Figure S2.png]

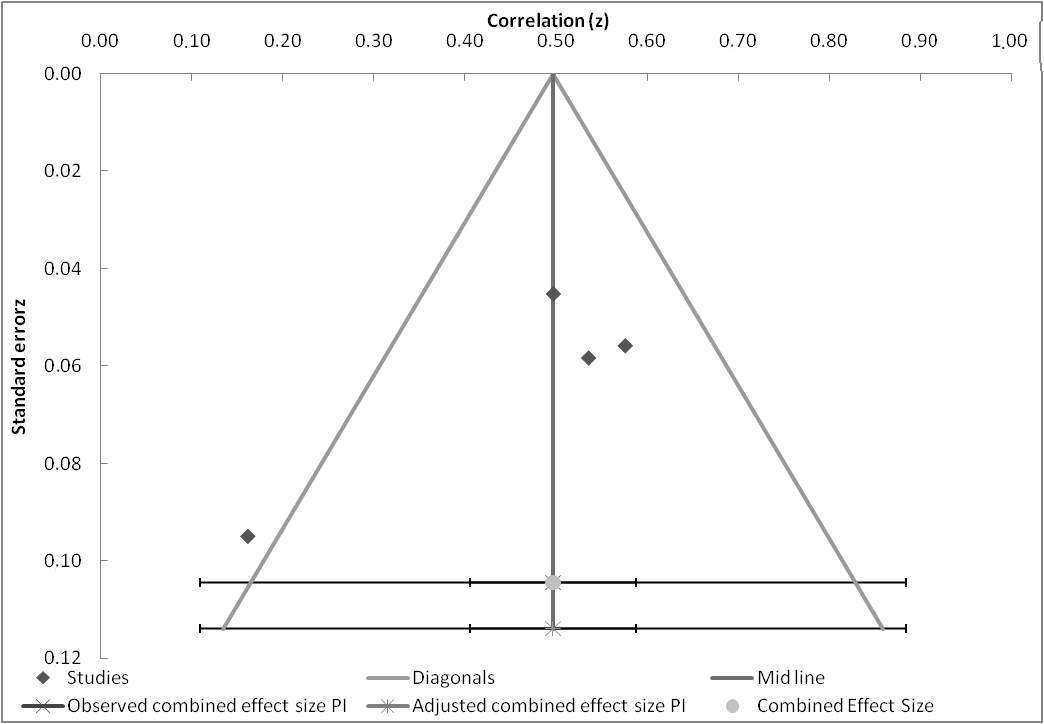

Supplement: Supplementary file 1 [file healthcare-12-00623-s001.zip › Supplementary Figure S3.png]

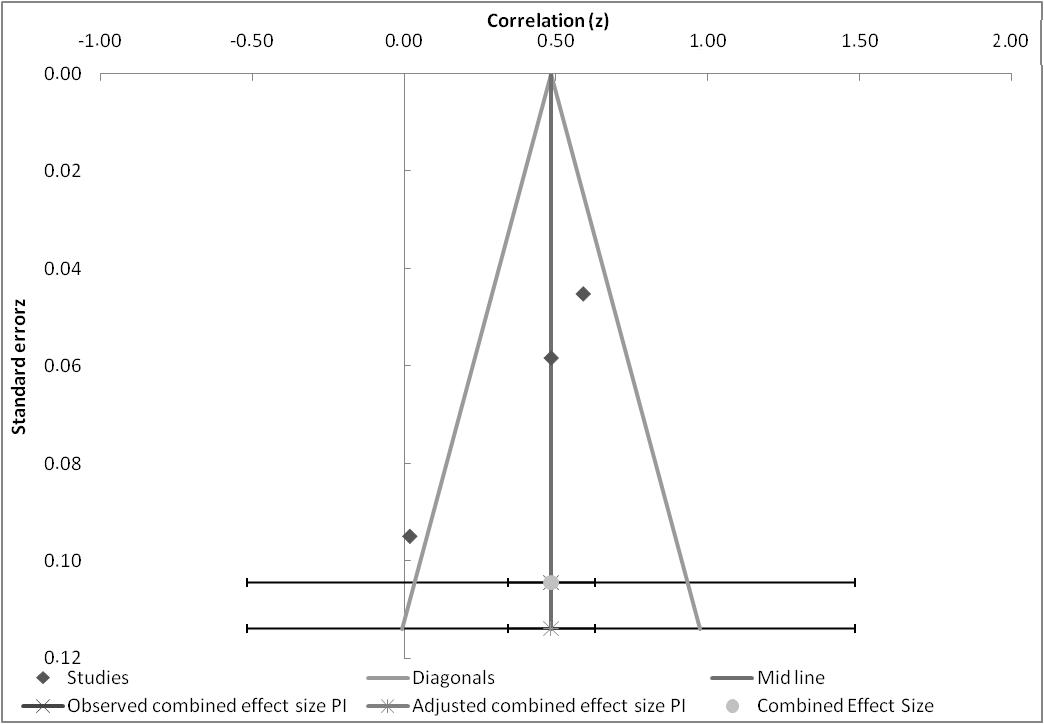

Supplement: Supplementary file 1 [file healthcare-12-00623-s001.zip › Supplementary Figure S4.png]
